# Buried Interface Regulation with TbCl_3_ for Highly-Efficient All-Inorganic Perovskite/Silicon Tandem Solar Cells

**DOI:** 10.1007/s40820-025-01763-8

**Published:** 2025-04-30

**Authors:** Wenming Chai, Weidong Zhu, He Xi, Dazheng Chen, Hang Dong, Long Zhou, Hailong You, Jincheng Zhang, Chunfu Zhang, Chunxiang Zhu, Yue Hao

**Affiliations:** 1 State Key Laboratory of Wide-Bandgap Semiconductor Devices and Integrated Technology, School of Microelectronics, Xidian University, Xi’an, 710071 People’s Republic of China; 2 Department of Electrical and Computer Engineering, National University of Singapore, 10 Kent Ridge Crescent, Singapore, 119260 Singapore; 3 Department of Nanomaterials, University of Melbourne, Parkville, Victoria 3010 Australia

**Keywords:** All-inorganic perovskite, Tandem solar cells, Interface regulation, Chloride passivation

## Abstract

All-inorganic perovskite materials exhibit exceptional thermal stability and promising candidates for tandem devices, while their application is still in the initial stage. Here, a metal halide doping strategy was implemented to enhance device performance and stability for inverted CsPbI_3_ perovskite solar cells (PSCs), which are ideal for integration into perovskite/silicon tandem solar cells. The lanthanide compound terbium chloride (TbCl_3_) was employed to improve buried interface between [4-(3,6-Dimethyl-9H-carbazol-9-yl) butyl] phosphonic acid (Me-4PACz) and perovskite layer, thereby enhancing the crystallinity of CsPbI_3_ films and passivating non-radiative recombination defects. Thus, the inverted CsPbI_3_ PSCs achieved an efficiency of 18.68% and demonstrated excellent stability against water and oxygen. Meanwhile, remarkable efficiencies of 29.40% and 25.44% were, respectively, achieved in four-terminal (4T) and two-terminal (2T) perovskite/silicon mechanically tandem devices, which are higher efficiencies among reported all-inorganic perovskite-based tandem solar cells. This study presents a novel approach for fabricating highly efficient and stable inverted all-inorganic PSCs and perovskite/silicon tandem solar cells.

**Supplementary Information:**

The online version contains supplementary material available at 10.1007/s40820-025-01763-8.

## Highlights

- The lanthanide compound of TbCl_3_ improved the wettability of Me-4PACz and further enhanced crystallization, in which the additional Cl^−^ ions passivate iodine vacancy and improve energy level alignment at buried interface.
- The inverted CsPbI_3_ PSCs with TbCl_3_ achieved a remarkable efficiency of 18.68% and enhanced stability in ambient air.
- Efficiencies of 29.40% and 25.44% were, respectively, achieved in 4T and 2T all-inorganic perovskite/silicon mechanically tandem devices.

**Supplementary Information:**

The online version contains supplementary material available at 10.1007/s40820-025-01763-8.

## Graphical abstract

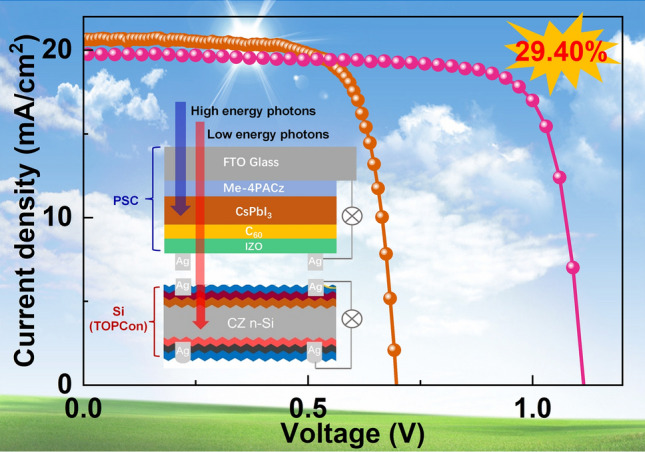

## Introduction

All-inorganic CsPbI_3−*x*_Br_*x*_ (0 ≤ x ≤ 3) perovskites demonstrate exceptional thermal stability (> 300 °C) and possess an adjustable optical bandgap ranging from 1.7 to 2.3 eV, offering significant potential for integration into tandem devices with silicon and organic solar cells [1–5]. The efficiency of single-junction CsPbI_3−*x*_Br_*x*_ perovskite solar cells (PSCs) has rapidly surpassed 22% [6–9]. However, the commonly used hole transport layer (HTL) of 2,2′,7,7′-tetrakis [N,N-di(4-methoxyphenyl)amino]-9,9′-spirobifluorene (spiro-OMeTAD) exhibits high parasitic absorption, which hinders its application in tandem devices. Additionally, the 4-tert-butylpyridine (t-BP) and Li-bis(trifluoromethanesulfonyl) imide (Li-TFSI) dopants could absorb moisture from the air, negatively impacting device stability [10–12]. Compared to the conventional structure, the electron transport layer (ETL) of inverted structure can protect the perovskite layer, exhibiting superior moisture stability and reduced hysteresis effects [13, 14]. Consequently, inverted PSCs have become a focal point of research in the photovoltaic field.

Inverted all-inorganic PSCs primarily utilize CsPbI_3_ and CsPbI_2_Br perovskite materials. Snaith et al. [15] first reported inverted CsPbI_3_ PSCs with an efficiency of 1.7%, which has since rapidly increased to achieve a record efficiency of 20.6% [16]. Compared to conventional structures, inverted all-inorganic PSCs exhibit inferior photovoltaic performance and larger open-circuit voltage (*V*_OC_) losses due to mismatched energy level alignment. Among common hole transport materials (HTMs), the hydrophilicity and acidity of PEDOT reduce interface stability, consequently decreasing the *V*_OC_ [17–19]. The wettability of precursor on the surfaces of poly[bis(4-phenyl) (2,4,6-trimethylphenyl) amine] (PTAA) is low, deteriorating the growth of perovskite films. The high annealing temperature restricts the application of organic HTLs in all-inorganic PSCs [20]. And, nickel oxide (NiO_x_) possesses low conductivity and high surface defects (Ni^3+^), which could hinder hole transfer, increase non-radiative recombination and accelerate perovskite degradation [14, 21]. Moreover, there is a detrimental reaction between NiO_x_ and dimethylammonium iodide (DMAI) in CsPbI_3_, which adversely affects device performance [22]. The energy mismatch between the all-inorganic perovskite film and the HTL/ETL also results in lower efficiency of inverted devices [19]. Tremendous efforts have been dedicated to optimizing the interface between perovskite and charge transport layers for synchronously enhanced PCE and stability of PSCs, such as bilayer homojunctions, ion-modulated radical doping [10–12, 23, 24]. Therefore, excellent charge transport materials can improve the crystallization of perovskite films and facilitate charge extraction at interfaces.

Ultra-thin self-assembled monolayers (SAMs) based on carbonyl units exhibit rich electron and high hole extraction capabilities, including [2-(3,6-dimethoxy-9H-carbazol-9-yl) ethyl] phosphonic acid (MeO-2PACz) and [4-(3,6-dimethyl-9H-carbazol-9-yl) butyl] phosphonic acid (Me-4PACz). The symmetric structure of carbonyl units results in a small dipole moment and enhanced charge transporting characteristics, as well as adjusts the work function of substrate [25, 26]. Currently, SAMs materials were commonly adopted to modify the interface between perovskite films and HTLs [22, 27–29]. Compared to other materials, Me-4PACz-based device exhibited the lower interface defects density (2 × 10^9^ cm^−2^) and one of highest efficiencies for single-junction and tandem solar cells [30]. However, the carbonyl units may also cause steric hindrance and wetting issues, which are detrimental to the density, uniformity, and crystallinity of perovskite films [31–33]. Therefore, optimizing the wettability of SAM layers can improve the crystallization of perovskite films and enhance the performance of inverted all-inorganic PSCs.

In this work, we utilized Me-4PACz with high charge carrier mobility, low affinity, and good photostability to fabricate inverted all-inorganic CsPbI_3_ PSCs. Terbium chloride (TbCl_3_) doping was employed to improve buried interface between Me-4PACz and perovskite, resulting in larger grains, smooth surface and enhanced phase stability of CsPbI_3_ films. Meanwhile, chloride passivation reduced defect density and suppressed non-radiative recombination at the buried interface. This also improved the energy alignment between the CsPbI_3_ film and Me-4PACz, thereby increasing the efficiency of inverted CsPbI_3_ PSCs to 18.68%. Additionally, the four-terminal (4T) and two-terminal (2T) perovskite/silicon mechanically tandem devices achieved the efficiencies of 29.40% and 25.44%, respectively. This provides a novel method for preparing efficient and stable all-inorganic CsPbI_3-x_Br_x_ PSCs and perovskite/silicon tandem devices, promoting the commercialization of perovskite photovoltaic technology.

## Experimental Section

### Materials and Reagents

Cesium iodide (CsI, 99.5%) and lead iodide (PbI_2_, 99.5%) were purchased from Alfa Aesar. Terbium trichloride (TbCl_3_, 99.5%), N,N-dimethylformamide (DMF, ≥ 99.9%), Dimethyl sulfoxide (DMSO, ≥ 99.9%), isopropyl alcohol (IPA, ≥ 99.9%), and chlorobenzene (CB, ≥ 99.9%) were purchased from Sigma-Aldrich. Dimethylammonium iodide (DMAI), Methylamine chloride (MACl), bathocuproine (BCP), C60, and (4-(3,6-dimethyl-9H-carbazole-9-yl)butyl) phosphonic acid (Me-4PACz, ≥ 99.9%) were purchased from Xi'an Yuri Solar Co., Ltd. All the above chemicals were used as received without any further purification.

### Solution Preparation

The 0.7 M CsPbI_3_ precursor was prepared by dissolving CsI, PbI_2_ and DMAI with the 1:1:1 molar ratio in the mixed solvent of DMF and DMSO (9:1). Then, the 1 mg mL^−1^ Me-4PACz and TbCl_3_ were mixed in ethyl alcohol. All solutions were stirred with magnetic stirrers for 12 h.

### Fabrication of CsPbI_3_ Single-Junction Devices

The FTO/glass substrates were ultrasonically cleaned sequentially with Decon90, deionized water, acetone, and ethanol for 15 min, respectively. The FTO/glass substrates were dried with N_2_ and treated with Ozone for 30 min. A Me-4PACz and TbCl_3_ mixed solvent was spin-coated on FTO at 5000 rpm for 30 s, followed by annealing for 10 min at 100 °C. The 80 μL CsPbI_3_ precursor was deposited on the Me-4PACz by spin-coating at 3000 rpm for 30 s. And, the CsPbI_3_ films were obtained by annealing at 200 °C for 5 min. Subsequently, 20 nm C60 and 7 nm BCP were sequentially deposited by vacuum evaporation under a vacuum pressure of 5 × 10⁻^4^ Pa. Finally, 100 nm silver (Ag) electrode was deposited with an active area of 0.07 cm^2^ by vacuum evaporation to obtain the inverted structure CsPbI_3_ PSCs.

### Fabrication of 2T Mechanically Tandem Devices

First, an 80 nm indium zinc oxide (IZO) was deposited on the surface of C60 using radio frequency (RF) magnetron sputtering, serving as a semi-transparent electrode. Next, 100 nm Ag grid electrode was thermally evaporated to enhance conductivity, forming semi-transparent PSCs. Finally, the prepared semi-transparent PSCs were affixed onto silicon solar cells and secured along the edges with high-temperature adhesive tape, completing the perovskite/silicon 2T mechanically stacked solar cell.

### Characterizations

SEM images were obtained by a Helios NanoLab G3 SEM. XPS and UPS spectra were acquired from X-ray photoelectron spectroscopy (Nexsa, Thermo Fisher). XRD was measured by an x’pert3 powder X-ray diffractometer (PANalytical, Netherlands). UV–vis absorption spectra were obtained by a spectrophotometer (U-4100, Hitachi). Steady-state PL and TRPL were tested on a FluoTime 300 spectrometer (PicoQuant, German). Light J-V curves were recorded from 1.3 to − 0.2 V with a step of 0.03 V s^−1^ by a Keithley 2450 source meter under a simulated AM 1.5Gillumination (100 mW cm^−2^), which was produced by an Oriel 92251A-1000 sunlight simulator. EQE was carried out on a 150 W xenon lamp (Oriel) equipped with a monochromator (Cornerstone 74,004). Dark *I-V* curves were obtained by Keithley 2636 source meter. EIS and Mott–Schottky were tested on an electrochemical workstation (CHI 660B) under dark conditions. The 1 V forward bias was applied to the EIS measurements. TPC and TPV were recorded by an oscilloscope, in which the sampling resistor of 50 Ω or 1 MΩ was applied. The photocurrent decay was measured under a 532 nm pulse laser (1000 Hz, 3.2 ns). The photovoltage decay was carried out under a 405 nm pulse laser (50 Hz, 20 ms). Contact angle was measured by OCA15EC with a precursor droplet of 5 μL.

## Results and Discussion

### Surface Chemistry of Perovskite Films with Buried Interface Regulation

**Fig. 1:**
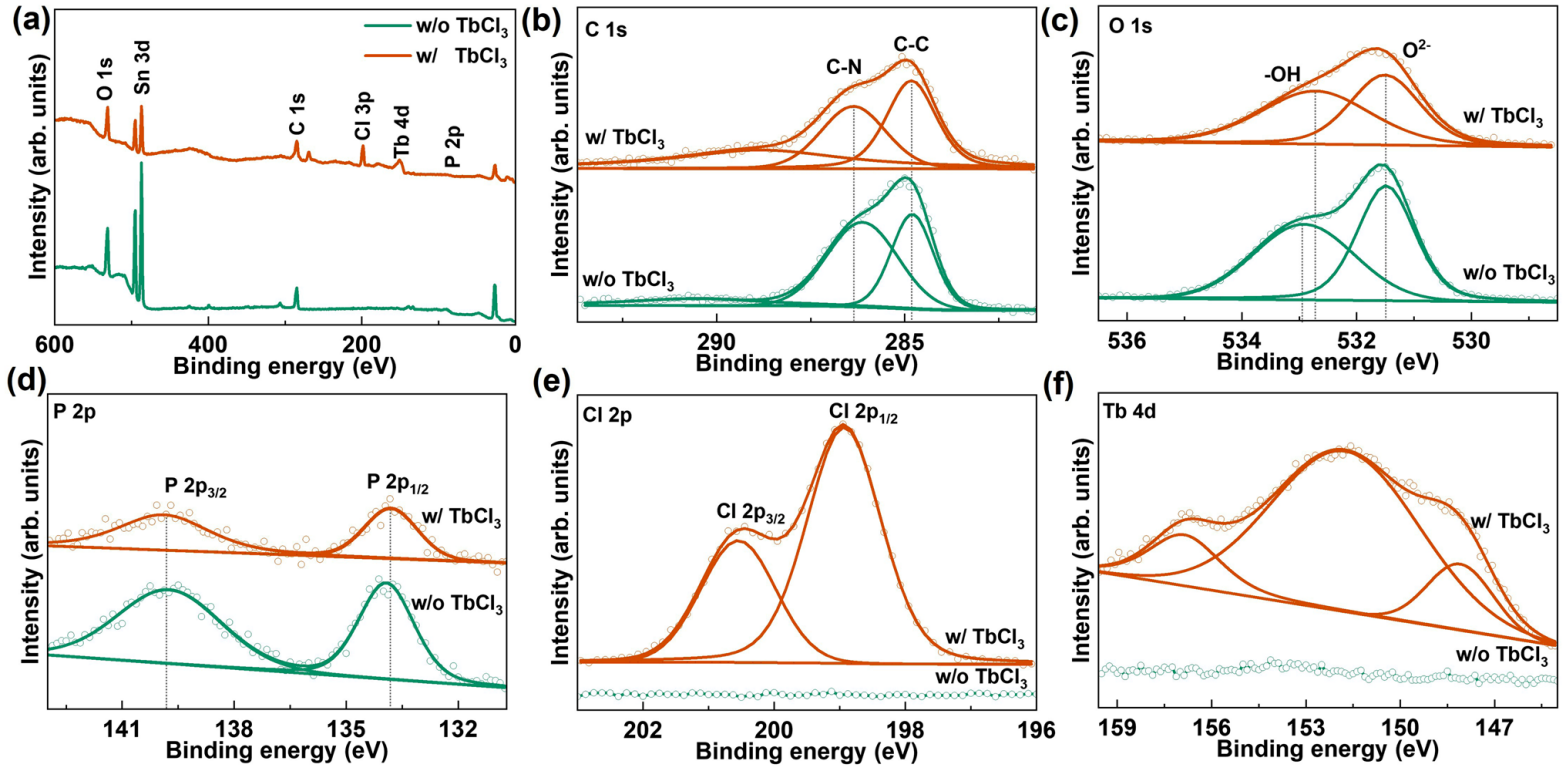
Surface chemistry of perovskite films. **a** XPS survey spectrum, **b** C 1*s*, **c** O 1*s*, **d** P 2*p*, **e** Cl 2*p*, and **f** Tb 4*d* core-level XPS spectra of the FTO/Me-4PACz substrate with and without TbCl_3_ doping

Me-4PACz is commonly used as an HTL to improve efficiency in both single-junction and perovskite/silicon tandem solar cells. Lanthanide ions (Ln^3+^) are employed to eliminate deep-level defects between the perovskite and HTL, thereby enhancing film quality [34, 35]. The doping state of TbCl_3_ in Me-4PACz was characterized using X-ray photoelectron spectroscopy (XPS). Then, XPS spectra were calibrated with the C 1*s* peak (284.8 eV). As shown in Fig. 1, the C 1*s*, O 1*s*, and P 2*p* spectra indicate that the Me-4PACz was successfully adhesion to the FTO substrate, while the Cl 2*p* and Tb 4*d* spectra confirm the incorporation of TbCl_3_ into Me-4PACz [36]. Compared to pure Me-4PACz on FTO, the OH- and P 2*p* XPS peaks shift toward higher binding energy in the sample with TbCl_3_ doping, indicating that the electrical properties were changed. During the annealing process, doped ions tend to diffuse from the buried interface to surface, thereby affecting the optoelectronic properties of the perovskite film. To investigate the impact of TbCl_3_ on the components, the surface composition of CsPbI_3_ films was characterized using XPS. As shown in Fig. S1, Tb 4*d* and Cl 2*p* were not detected in CsPbI_3_ films based on TbCl_3_-doped Me-4PACz, indicating that Tb^3+^ and Cl^−^ did not diffuse to the surface. The Pb 4*f* and Cs 3*d* XPS peaks shifted to higher binding energy, whereas the I 3*d* shifted to lower binding energy. The difference in ionic radii leads to distortion in the BX_6_ (B = Pb/Tb, X = I/Cl) octahedral structure, thus altering the surface chemical characteristics [28, 29]. These shifts are primarily attributed to the incorporation of Tb^3+^ and Cl^−^ into the CsPbI_3_ crystal structure, where they, respectively, occupied Pb^2+^ and I^−^ sites. Additionally, excessive Cl^−^ can passivate V_I_ defects at buried interface, thereby enhancing the device optoelectronic properties.

### Improved Morphology and Crystal Quality of Buried Interface Regulation with TbCl_3_

**Fig. 2:**
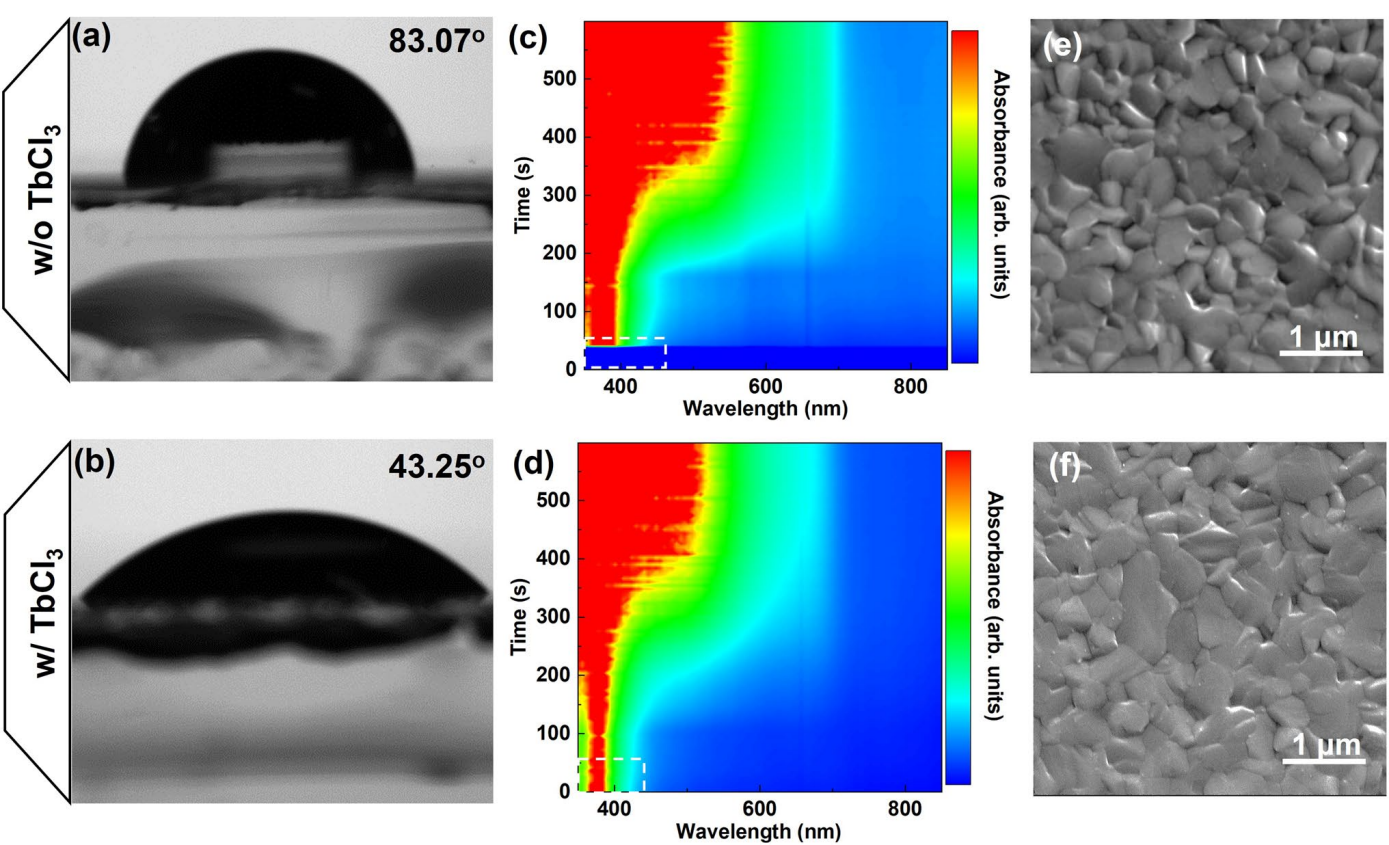
Crystallization process and Morphology of perovskite films. Contact angles of the CsPbI_3_ precursor solution on the surface of Me-4PACz **a** without and **b** with TbCl_3_ doping, respectively. In situ absorption spectra of CsPbI_3_ intermediate phase films based on Me-4PACz **c** without and **d** with TbCl_3_ doping during the annealing process, respectively. SEM images of CsPbI_3_ films deposited on Me-4PACz **e** without and **f** with TbCl_3_ doping

The non-polar groups of long alkyl chains (C_4_H_8_) in Me-4PACz causes high hydrophobicity, resulting in poor coverage of the perovskite film. Metal ion have commonly been used to improve the wettability of the substrate [32, 37]. The effect of TbCl_3_ on the wettability of Me-4PACz was performed using contact angle measurements. As shown in Fig. 2a, b, TbCl_3_ doping significantly reduced the contact angle of the CsPbI_3_ precursor solution on substrate from 83.07° to 43.25°. Compared to Me-4PACz, the lower contact angle on TbCl_3_-doped Me-4PACz facilitates the spreading of the precursor solution, leading to fully covered and uniform perovskite film. This is contributed to enhance the crystallization and reduce surface roughness of the CsPbI_3_ perovskite film. To investigate the influence of TbCl_3_ doping on the crystallization process, in situ absorption spectroscopy was used to characterize the crystallization of CsPbI_3_ films during the annealing process. As shown in Fig. 2c, d, the CsPbI_3_ film without TbCl_3_ doping shows no obvious absorption within the first 50 s, whereas the CsPbI_3_ film based on TbCl_3_-doped Me-4PACz exhibits strong absorption in the short-wavelength region at the beginning. After approximately 250 s, the absorption edges of both films show no significant changes, indicating that TbCl_3_ can accelerate the phase transition process of the CsPbI_3_ film [38]. As shown in Fig. 2e, f, TbCl_3_ doping in Me-4PACz significantly enhances the crystallization quality in comparison with the control sample, resulting in CsPbI_3_ films with larger grain sizes and uniform distribution. Moreover, the cross sectional SEM images are presented in Fig. S2, in which the control sample exhibits pinholes at interface between FTO and perovskite film. However, the CsPbI_3_ film with TbCl_3_ does not have obvious pinholes at interface due to the reduced contact angle of precursor on Me-4PACz, which is conducive to enhance the *V*_OC_ and short-circuit current density (*J*_SC_) of devices. And, the atomic force microscopy (AFM) images are shown in Fig. S3, the CsPbI_3_ film with TbCl_3_ doping has a lower root mean square (RMS) roughness of 19.0 nm in comparison with the control samples of 24.8 nm, similar to the SEM results. Therefore, TbCl_3_ improves the wettability of Me-4PACz and accelerates the phase transition rate, forming CsPbI_3_ films with great morphology. This can enhance the interface contact between the perovskite films and ETL, contributing to the improved optoelectronic performance.

**Fig. 3:**
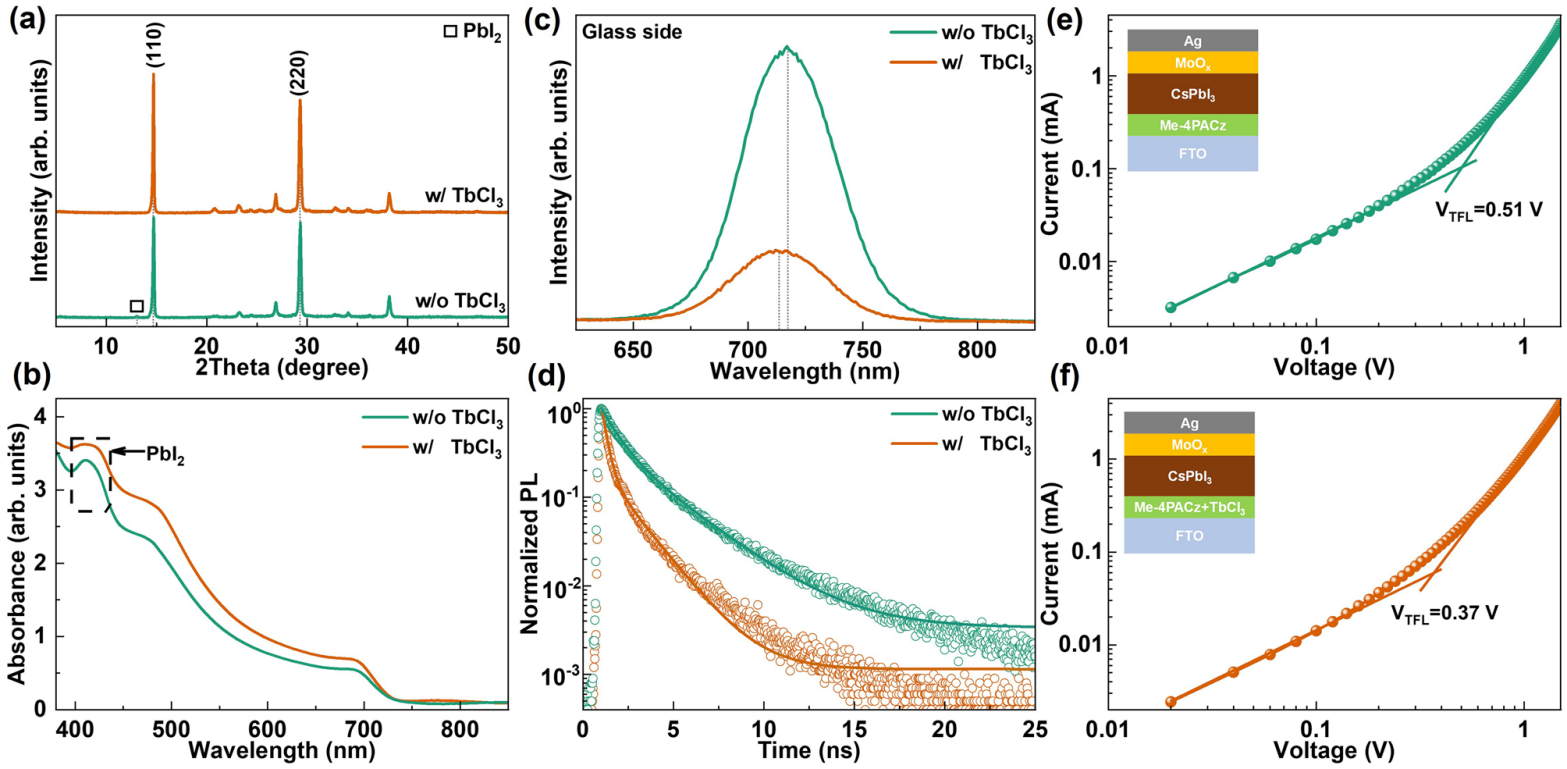
**a** XRD patterns, **b** UV–vis absorption spectra, **c** steady-state PL spectra, and **d** TRPL curves of CsPbI_3_ films on Me-4PACz with and without TbCl_3_ doping, respectively. **e**, **f** SCLC curves of CsPbI_3_ films based on Me-4PACz without and with TbCl_3_ doping

To further investigate the effect of TbCl_3_ on the CsPbI_3_ films, X-ray diffraction (XRD), ultraviolet–visible spectroscopy (UV–vis), photoluminescence (PL), time-resolution photoluminescence (TRPL), and space-charge-limited current (SCLC) were used to characterize the optoelectronic properties of the perovskite films. As shown in Fig. 3a, there are two diffraction peaks at 14.71° and 29.28°, corresponding to the (110) and (220) crystal planes of γ-CsPbI_3_ [7–9]. Compared to the control sample, the CsPbI_3_ film based on TbCl_3_-doped Me-4PACz exhibits stronger diffraction peaks, indicating that TbCl_3_ significantly enhances the crystallinity of the perovskite film, which is consistent with the observed SEM results. The control sample shows a small XRD peak at 13.09°, corresponding to excessive PbI_2_. However, the CsPbI_3_ film prepared with TbCl_3_-doped Me-4PACz does not exhibits PbI_2_ peak, indicating that TbCl_3_ suppresses the formation of excessive PbI_2_ [39]. As presented in Fig. S4, the (110) and (220) peaks of the CsPbI_3_ film prepared with TbCl_3_-doped Me-4PACz shift to smaller angles in comparison with the control sample. This indicates that the lattice constant was reduced according to Bragg's law, proving that the smaller ionic radii of Tb^3+^ and Cl^−^ are incorporated into the CsPbI_3_ lattice [28, 40, 41]. Ions doping leads to chemical changes in CsPbI_3_ film with TbCl_3_, as observed in XPS results. As shown in Fig. 3b, both samples have similar absorption onsets (~ 725 nm), corresponding to an optical bandgap of 1.71 eV. Similar to the XRD results, the CsPbI_3_ film with TbCl_3_ exhibits higher absorbance intensity, indicating that TbCl_3_ enhances the crystallinity of the perovskite film. The control sample shows an exciton absorption peak at 410 nm, according to reported literature, which corresponds to excess PbI_2_ components [42, 43]. Therefore, TbCl_3_ doping in Me-4PACz significantly enhances the crystallinity of CsPbI_3_ films and suppresses the formation of excess PbI_2_.

The interface property was performed using PL results obtained from the surface and glass substrate sides, respectively. As presented in Fig. S5, both samples exhibit similar PL peaks at 725 nm, consistent with the aforementioned absorption results. The CsPbI_3_ film prepared with TbCl_3_-doped Me-4PACz shows higher PL intensity and blue shift in comparison with control sample, attributed to TbCl_3_ enhancing the crystallinity of the CsPbI_3_ film and reducing defect density. The PL results obtained from the glass substrate side were used to investigate the buried interface between the perovskite film and Me-4PACz. As shown in Fig. 3c, the CsPbI_3_ film prepared with TbCl_3_-doped Me-4PACz exhibits a lower PL intensity and blue shift, indicating that TbCl_3_ incorporated into the lattice forms wide-bandgap perovskite and improves carrier extraction at the buried interface [44]. Similar results can be also derived from TRPL in Fig. 3d, the CsPbI_3_ film prepared with TbCl_3_-doped Me-4PACz has a faster PL decay compared to the control sample. As displayed in Table S1, the fast decay part τ_1_ decreases from 0.46 to 0.28 ns, and the slow decay part increases from 1.66 to 2.79 ns after TbCl_3_ modification. As a result, the average lifetime decreased from 2.67 to 1.55 ns after modifying with TbCl_3_. Smaller τ_1_ and larger τ_2_ value indicate the rapid extraction of charge and the reduction of defect state density at the modified perovskite buried interface. Based on the SCLC theory, the defect densities of CsPbI_3_ films with and without TbCl_3_ were analyzed. Figure 3e, f shows the *J-V* curves of single-hole devices, with the device structure illustrated in the inset. They display ohmic behavior at low bias, accompanied by a nonlinear increase in current density as the voltage exceeds trap-filled limit voltage (*V*_TFL_), which is directly proportional to the trap-state density (n_t_) according to the following equation [7]:

$${\text{n}}_{\text{t}}=\frac{2{\text{V}}_{\text{TFL}}{\upvarepsilon }_{0}{\upvarepsilon }_{\text{r}}}{{\text{eL}}^{2}}$$

where the e is the elementary charge (e = 1.6 × 10^−19^ C), *ε*_*0*_ and *ε*_*r*_ are vacuum permittivity (ε_0_ = 8.85 × 10^−12^ F m^−1^) and relative permittivity (ε_r_ = 6.32), L is the thickness of perovskite film (L = 500 nm), respectively [45, 46]. As indicated, the CsPbI_3_ films prepared with TbCl_3_-doped Me-4PACz obtains a lower *V*_TFL_ of 0.37 V than the control device of 0.51 V, leading to a lower of 1.035 × 10^15^ vs. 1.427 × 10^15^ cm^−3^. Based on the above results, TbCl_3_ enhances the crystallinity and reduces defect density of CsPbI_3_ films, as well as improves carrier extraction capability at the buried interface, contributing to enhanced device performance.

### Performance of Inverted CsPbI_3_ Single-Junction Device

**Fig. 4:**
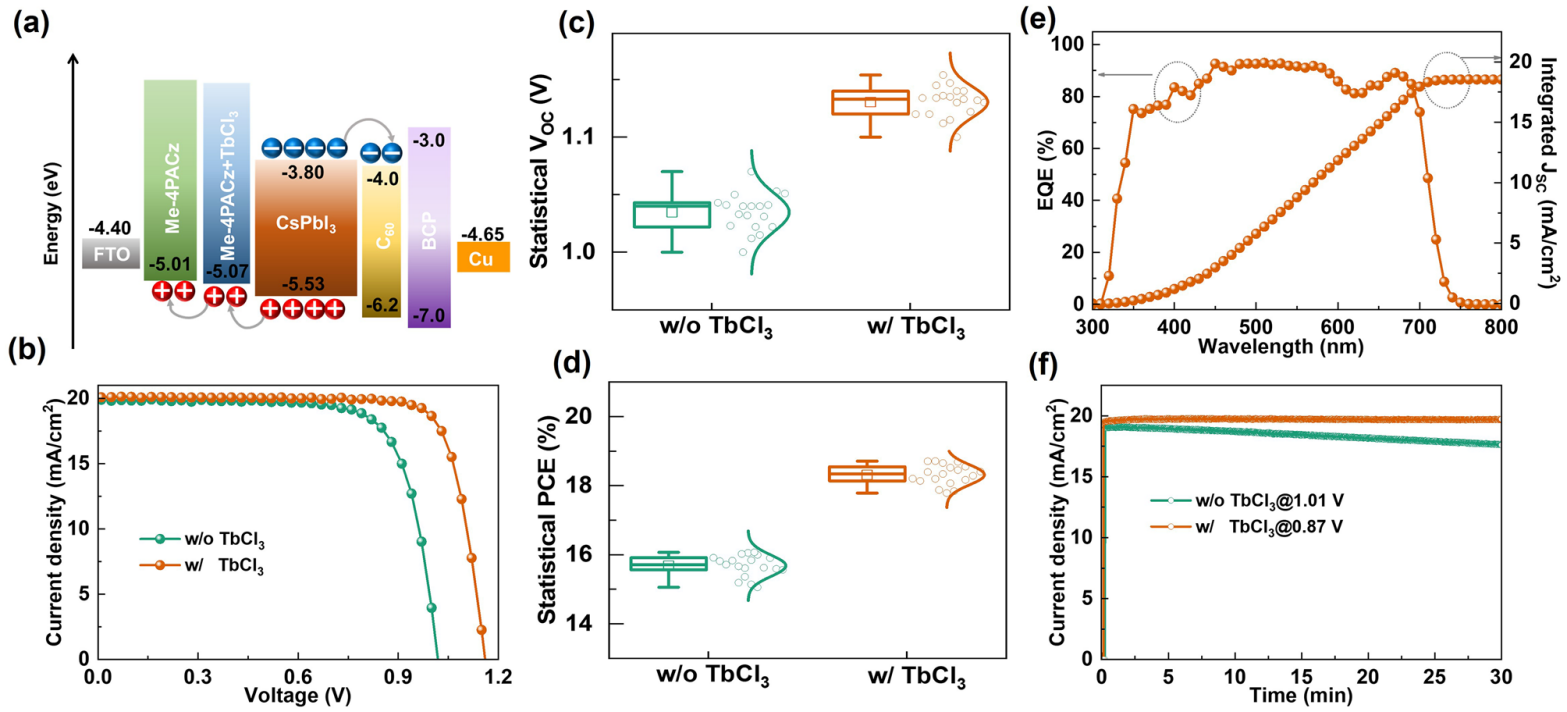
Influence of TbCl_3_ on PSCs performance. **a** Energy band alignment diagram of CsPbI_3_ PSCs based on Me-4PACz with and without TbCl_3_ doping. **b** *J–V* curves of the CsPbI_3_ PSCs with varying TbCl_3_ concentrations. **c** Light *J-V* curves and **d** Statistical PCE of CsPbI_3_ PSCs based on Me-4PACz with and without TbCl_3_ doping, respectively. **e** EQE spectra and integrated current curves of CsPbI_3_ PSCs based on Me-4PACz. **f** Steady-state output curves at the maximum power point of CsPbI_3_ PSCs based on Me-4PACz with and without TbCl_3_ doping

To investigate the effect of TbCl_3_ doping on the photovoltaic performance, we fabricated inverted CsPbI_3_ PSCs based on the device structure of FTO/Me-4PACz/CsPbI_3_/C60/BCP/Cu. The surface work function and energy band alignment of Me-4PACz were measured using ultraviolet photoelectron spectroscopy (UPS). As shown in Fig. S6, TbCl_3_ doping reduces the work function of Me-4PACz from 4.18 to 4.01 eV, with corresponding E_VBM_ values of − 5.01 and − 5.07 eV, respectively. Using the reported energy band values of other materials, the energy band alignment of CsPbI_3_ PSCs was obtained [47]. As shown in Fig. 4a, TbCl_3_ reduces the VBM difference between CsPbI_3_ films and Me-4PACz, facilitating hole extraction and transport, thereby improving the *V*_OC_ and PCE of the device [44, 48–50]. Figure 4b shows the current density–voltage (J-V) curves of devices with different amounts of TbCl_3_ dopants, and the photovoltaic parameters are statistically distributed in Fig. S7, in which the optimal concentration is 1 mg mL^−1^ and used in other characterizations. As shown in Fig. 4c and Table S2, the control sample exhibits a maximum PCE of 15.34%, with a *V*_OC_ of 1.017 V, fill-factor (FF) of 0.758, and *J*_SC_ of 19.90 mA cm^−2^. For the CsPbI_3_ PSCs prepared with TbCl_3_-doped Me-4PACz, the PCE increases to 18.68%, with a *V*_OC_ of 1.162 V and FF of 0.799. As the *J-V* curves of the CsPbI_3_ PSC under forward and reverse scan are shown in Fig. S8, the device fabricated with TbCl_3_-doped Me-4PACz generates less hysteresis. Due to the improved crystallization, the CsPbI_3_ film fabricated with TbCl_3_-doped Me-4PACz exhibits few ions migration and non-radiative recombination near interfaces. Moreover, the TbCl_3_ doping decreases dark current of CsPbI_3_ PSCs, as provided in Fig. S9, which revealed that the leakage channel is suppressed to reduce the non-radiative recombination. The remarkable enhancement in these photovoltaic performance parameters should be ascribed to the improved optoelectronic properties of CsPbI_3_ film based on Me-4PACz with TbCl_3_.

**Fig. 5:**
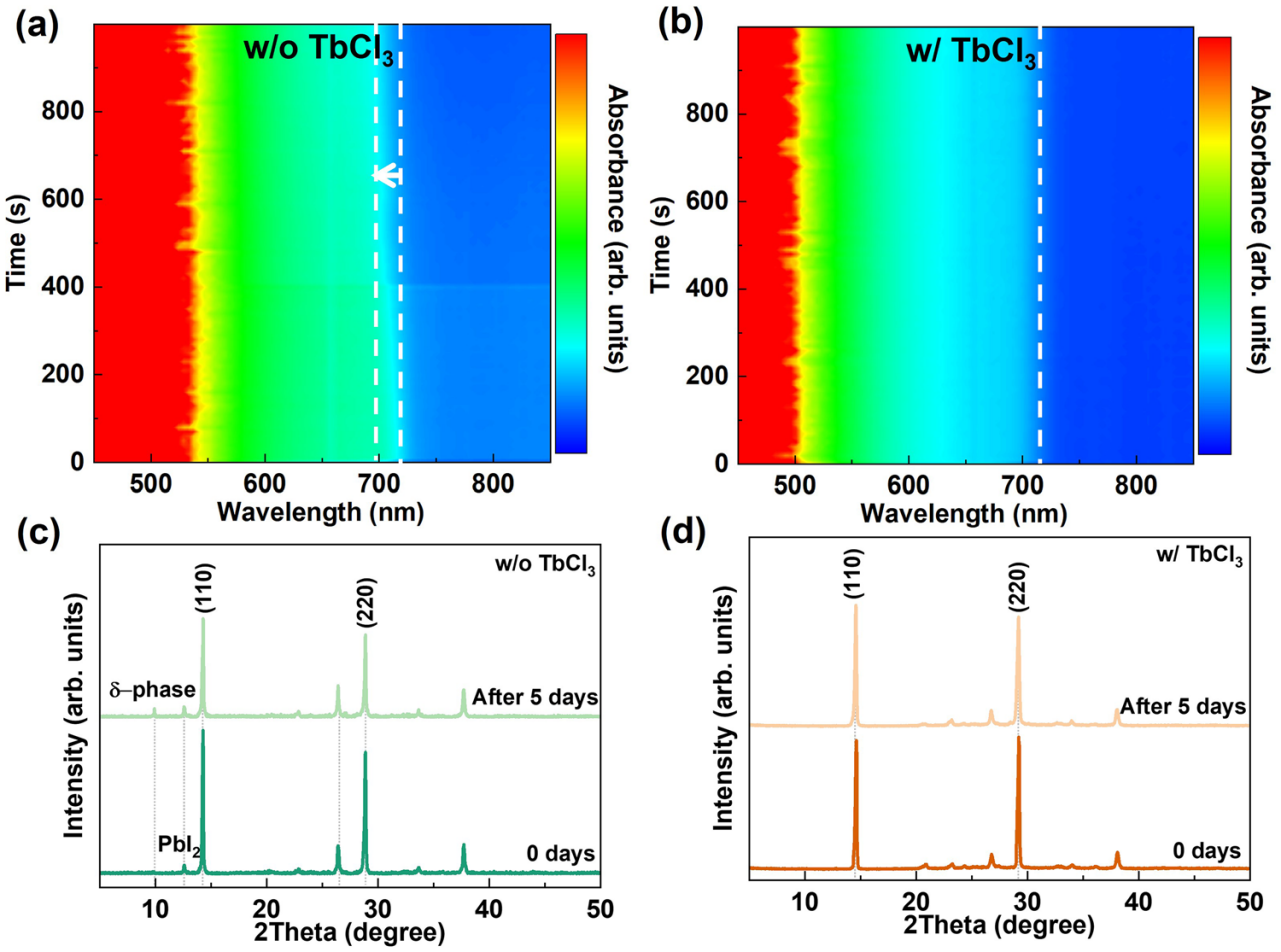
In situ absorption spectra of CsPbI_3_ films prepared on Me-4PACz **a** without and **b** with TbCl_3_ doping in air environment, respectively. XRD patterns of CsPbI_3_ films prepared **c** without and **d** with TbCl_3_-doped Me-4PACz after storing for 5 days in ambient air

The photovoltaic parameters of 20 independent devices were statistically analyzed, as presented in Figs. 4d and S10. Compared to the control sample, the CsPbI_3_ PSCs prepared with TbCl_3_-doped Me-4PACz exhibit higher average *V*_OC_, FF and PCE values. This is consistent with the trend observed in the *J-V* results, indicating that the TbCl_3_-doped Me-4PACz method has good reproducibility. Figure 4e shows the EQE spectra of the CsPbI_3_ PSCs with a photo-response cut-off edge at ~ 725 nm, which is consistent with the absorption results. The integrated current is 18.60 mA cm^−2^, closing to the *J*_SC_ value of the device. Moreover, the steady-state output current density at the maximum power point was employed to evaluate the operational stability, as shown in Fig. 4f. Compared to the control sample, the CsPbI_3_ PSC prepared with TbCl_3_-doped Me-4PACz maintains a constant photocurrent density after 30 min under continuous illumination. In situ absorption spectroscopy was used to perform the effect of TbCl_3_ on the stability of CsPbI_3_ films in ambient air. As shown in Fig. 5, the absorption onset of the control sample gradually blue-shifts, while that of the CsPbI_3_ film prepared with TbCl_3_-doped Me-4PACz remains almost unchanged after 1000 s. This indicates that TbCl_3_ significantly enhances the water and oxygen stability of the perovskite film [41, 51]. Moreover, as depicted in Fig. 5c, d, we measured the XRD patterns of unencapsulated CsPbI_3_ films after storing in ambient air. The intensity of control CsPbI_3_ films is reduced after storing for 5 days, and forming an additional peak at 10.2° corresponding to the δ-phase. In comparison, the CsPbI_3_ films prepared with TbCl_3_-doped Me-4PACz does not decrease the intensity and form additional peaks. Furthermore, the PL intensity of CsPbI_3_ films without TbCl_3_ is reduced after storing for 5 days, as shown in Fig. S11, whereas it does not change in the CsPbI_3_ films prepared with TbCl_3_-doped Me-4PACz. The δ-phase can result in enhanced non-radiative recombination, thereby reduce *V*_OC_ and PCE over time, as shown in Fig. S12**.** Overall, TbCl_3_ passivates interfacial defects and improves energy band alignment, meanwhile enhancing the photovoltaic performance and stability of CsPbI_3_ PSCs.

Carrier dynamics of CsPbI_3_ PSCs were thoroughly investigated using transient photocurrent (TPC)/transient photovoltage (TPV), electrochemical impedance spectroscopy (EIS), and Mott–Schottky (M-S) characterizations. Figure S13a, b displays the TPC and TPV curves, fitted with a single exponential decay function. The CsPbI_3_ PSCs prepared with TbCl_3_-doped Me-4PACz exhibit faster photocurrent decay (τ_ave_ = 0.59 μs) and slower photovoltage decay (τ_ave_ = 0.031 ms), indicating that TbCl_3_ enhances carrier extraction and reduces carrier recombination [52, 53]. This also well support the results of PL and TRPL. As shown in Fig. S14a, CsPbI_3_ PSCs prepared with TbCl_3_-doped Me-4PACz improved recombination resistance (*R*_rec_) from 2069 to 3198 Ω in comparison with the control sample, indicating that the carrier recombination is suppressed at the Me-4PACz/CsPbI_3_ interface [29, 54]. From the *M-S* plot in Fig. S14b, the built-in voltage (*V*_bi_) values for CsPbI_3_ PSCs without and with TbCl_3_ doping were fitted to be 1.05 and 1.19 V, respectively. A relatively larger *V*_bi_ implies stronger driving force for carrier transport and wider depletion region to reduce carrier recombination, which is contributed to improve the V_OC_ of CsPbI_3_ PSCs as JV results [44, 55]. Ultimately, the performance improvement of CsPbI_3_ PSCs is attributed to the beneficial characteristics of CsPbI_3_ films prepared with TbCl_3_-doped Me-4PACz, including larger grain size, high crystallinity, low defect density, and improved energy band alignment.

### Performance of All-Inorganic Perovskite/Silicon Tandem Device

**Fig. 6:**
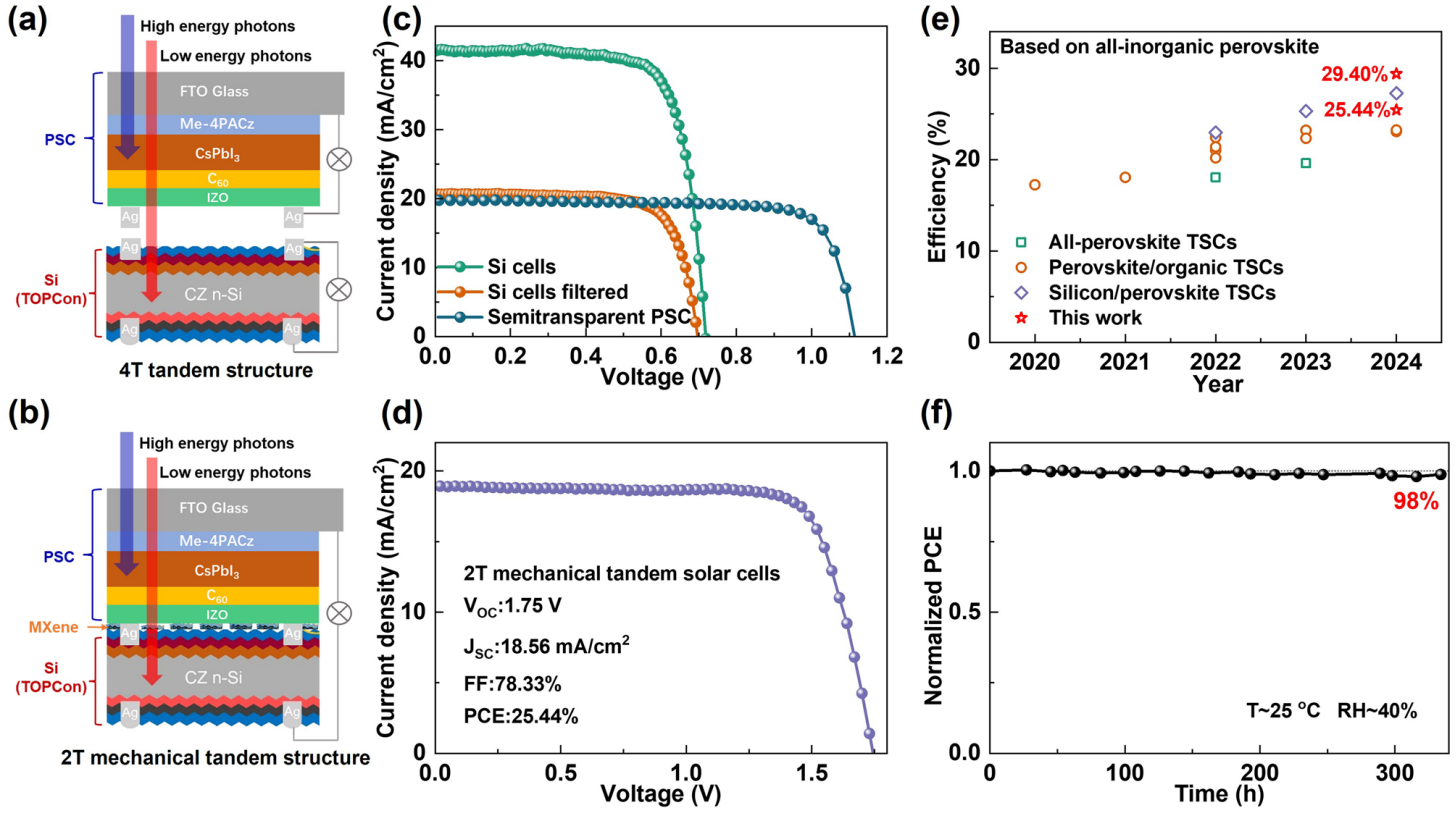
Performance and stability of all-inorganic perovskite/silicon tandem devices. **a, b** Schematic diagram of the 4T and 2T mechanically tandem device structure based on the inverted CsPbI_3_ PSC and silicon solar cell. **c** *J-V* curves of semi-transparent CsPbI_3_ PSC, silicon solar cell before and after shading. **d** *J-V* curves of the 2T perovskite/silicon mechanically tandem device. **e** Summary of tandem device based on all-inorganic perovskite solar cells. **f** Storage stability of the two-terminal tandem device in an air environment with RH ~ 40%

There are two basic structures for perovskite/silicon tandem solar cells. The 4T tandem structure has no specific requirements for the bandgap of the top cell, and the 2T tandem structure requires the top cell with bandgap of ~ 1.7 eV. Therefore, wide-bandgap inverted CsPbI_3_ PSCs are suitable as the top cell in tandem devices [56, 57]. As shown in Fig. 6a, semi-transparent CsPbI_3_ PSCs were fabricated using IZO as the transparent electrode. As shown in Fig. 6c and Table S3, the optimal efficiency of semi-transparent inverted CsPbI_3_ PSCs is 17.57%. Compared to the metal electrode, *V*_OC_ of semi-transparent device is reduced because of the damage to C60 during the IZO sputtering process. The small-area (2 × 2.5 cm^2^) silicon solar cell has an efficiency of 11.83% after shading with the semi-transparent CsPbI_3_ PSCs. Therefore, the 4T all-inorganic CsPbI_3_ perovskite/silicon tandem solar cells obtained a remarkable PCE of 29.40% by simply adding the efficiencies of two sub-cells. Moreover, as presented in Fig. 6b, a mechanically stacked 2T perovskite/silicon tandem solar cell was fabricated using MXene with high transmittance and conductivity as the interconnection layer, referenced to our previous work [58, 59]. As shown in Fig. 6d, the 2T mechanically tandem devices achieved an optimal efficiency of 25.44% with *V*_OC_ of 1.75 V, FF of 0.783, and *J*_SC_ of 18.56 mA cm^−2^, which is one of high efficiencies of all-inorganic perovskite tandem solar cells. The photovoltaic parameters of the previously reported perovskite/silicon tandem solar cells based on inorganic perovskite are summarized in Table S4 and the results of the corresponding PCE distributions are shown in Fig. 6e. As shown in Fig. 6f, after storage in an air environment with RH ~ 20% for 320 h, the 2T tandem cell based on inverted CsPbI_3_ PSCs retained 98% of its initial efficiency, demonstrating excellent stability. Therefore, inverted CsPbI_3_ PSCs show great potential for application in tandem devices.

## Conclusion

In conclusion, the lanthanide compound TbCl_3_ doping was employed to improve the wettability and optoelectronic properties of Me-4PACz, enhancing the crystallization of CsPbI_3_ films with large grains and smooth surface. The Tb^3+^ and Cl^−^ could diffuse into the CsPbI_3_ perovskite lattice, enhancing the stability of black phase. Moreover, the excessive Cl^−^ passivates the V_I_ defects at the buried interface, suppressing non-radiative recombination loss and enhancing the V_OC_. Meanwhile, TbCl_3_ improved the energy band alignment between the CsPbI_3_ film and Me-4PACz, enhancing the efficiency of the inverted CsPbI_3_ PSCs to 18.68% and exhibiting excellent stability. Meantime, the CsPbI_3_ PSCs were used to fabricate 4T and 2T perovskite/silicon mechanically tandem devices, achieving efficiencies of 29.40% and 25.44%, respectively. Therefore, this provides a novel method for preparing highly efficient and stable all-inorganic PSCs and perovskite/silicon tandem solar cells, and accelerating the commercialization of PSCs.

## Supplementary Information

Below is the link to the electronic supplementary material.

Supplementary file1 (DOCX 1766 KB)
