# Supplementary material for: Buried Interface Regulation with TbCl3 for Highly-Efficient All-Inorganic Perovskite/Silicon Tandem Solar Cells
Source: Nanomicro Lett. 2025 Apr 30;17:244. doi: 10.1007/s40820-025-01763-8 (PMC12043559; doi:10.1007/s40820-025-01763-8)
Supplement: Supplementary file 1 — Supplementary file1 (DOCX 1766 KB) [file 40820_2025_1763_MOESM1_ESM.docx]

Supporting Information for

**Buried Interface Regulation with TbCl_3_ for Highly-Efficient All-Inorganic Perovskite/Silicon Tandem Solar Cells**

Wenming Chai^1^, Weidong Zhu^1^*, He Xi^1,3^, Dazheng Chen^1^, Hang Dong^1^, Long Zhou^1^, Hailong You^1^, Jincheng Zhang^1^, Chunfu Zhang^1^*, Chunxiang Zhu^2^ and Yue Hao^1^

^1^State Key Laboratory of Wide-Bandgap Semiconductor Devices and Integrated Technology, School of Microelectronics, Xidian University, Xi'an 710071, P. R. China

^2^Department of Electrical and Computer Engineering, National University of Singapore, 10 Kent Ridge Crescent, 119260, Singapore

^3^Department of nanomaterials, University of Melbourne, Parkville Victoria, 3010, Australia

*Corresponding authors. E-mail: [wdzhu@xidian.edu.cn](mailto:wdzhu@xidian.edu.cn) (Weidong Zhu); [cfzhang@xidian.edu.cn](mailto:cfzhang@xidian.edu.cn) (Chunfu Zhang)

**Supplementary Figures and Tables**


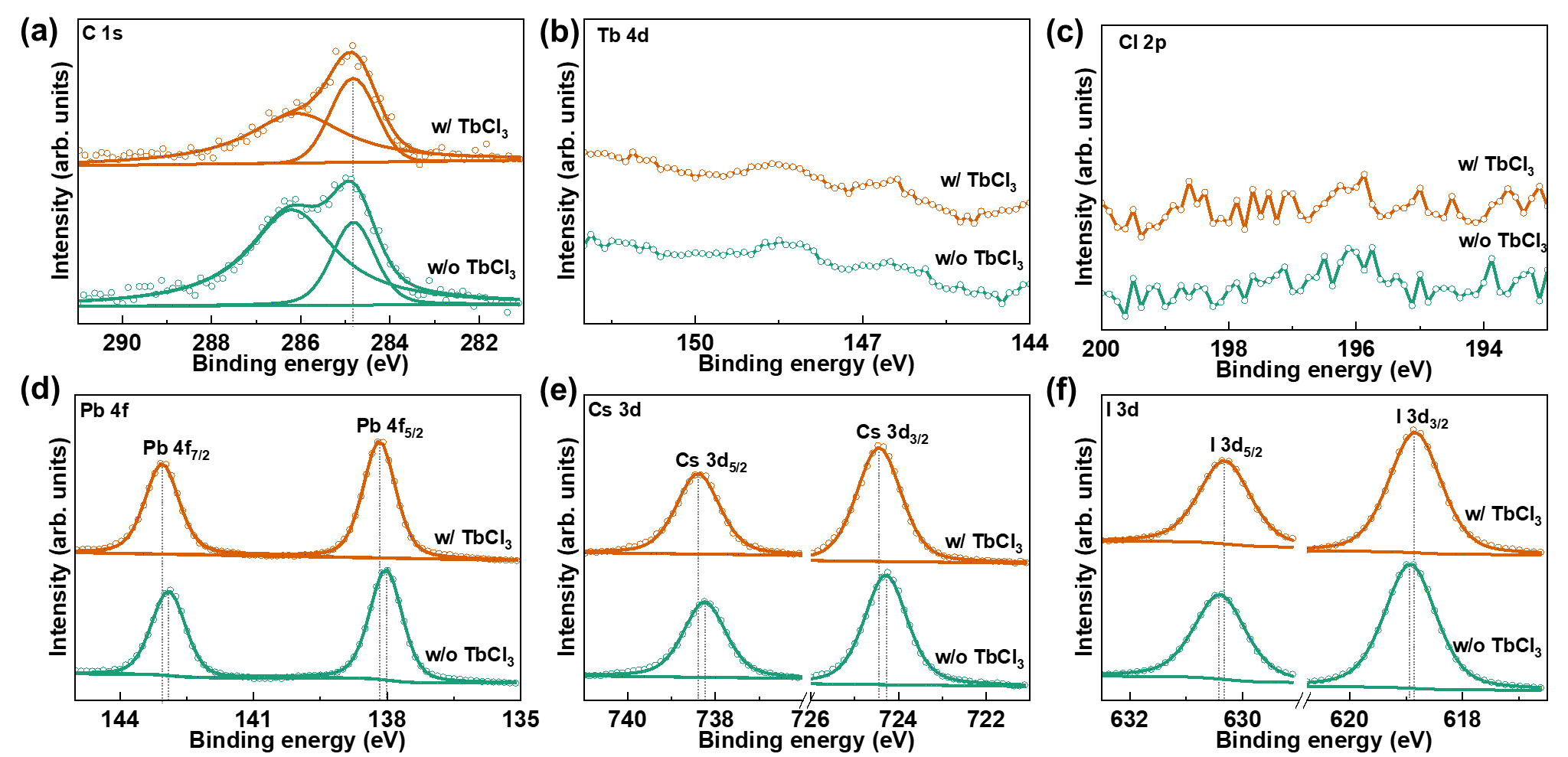


**Fig. S1** **a** C 1s, **b** Tb 4d, **c** Cl 2p, **d** Pb 4f, **e** Cs 3d, and **f** I 3d core-level XPS spectra of CsPbI_3_ films based on Me-4PACz with and without TbCl_3_ doping


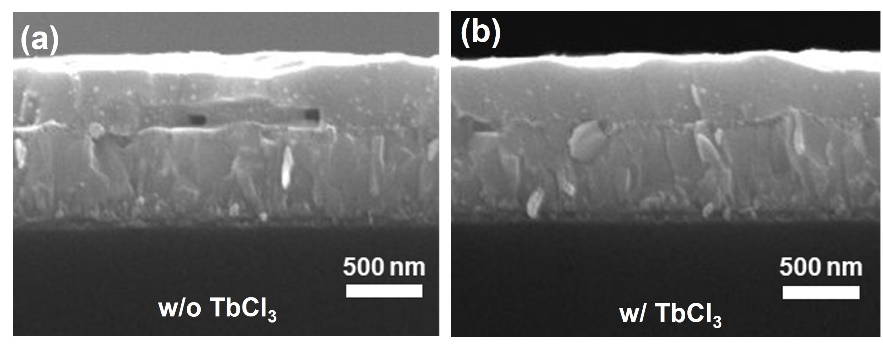


**Fig. S2** Cross-section SEM images of the CsPbI_3_ films based on Me-4PACz **a** without and **b** with TbCl_3_ doping

**
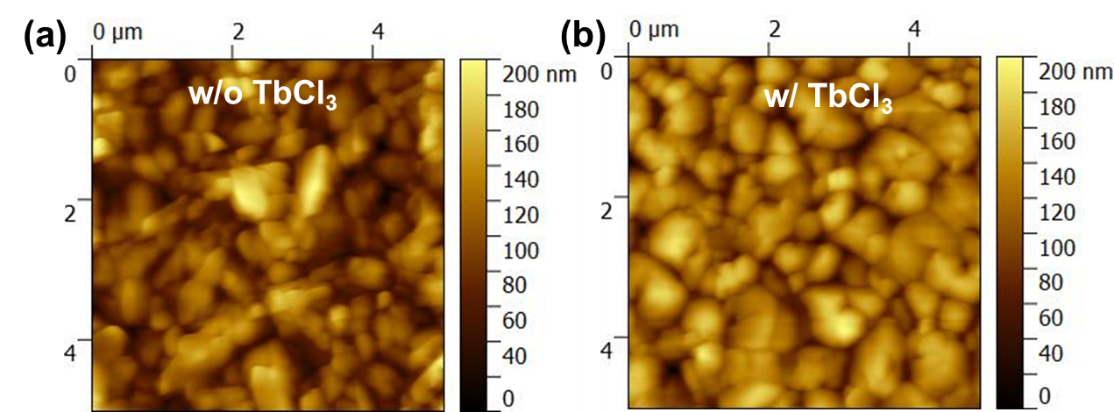
**

**Fig. S3** AFM images of the CsPbI_3_ films based on Me-4PACz **a** without and **b** with TbCl_3_ doping


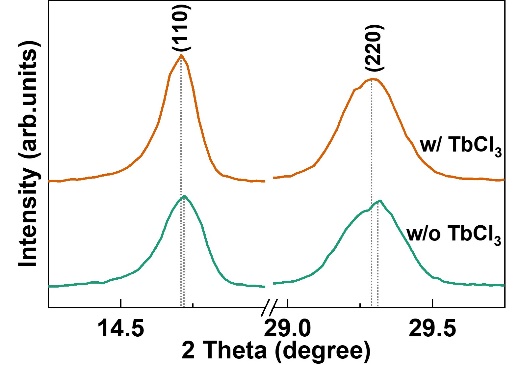


**Fig. S4** Magnified (110) and (220) XRD peaks of CsPbI_3_ films prepared on Me-4PACz with and without TbCl_3_ doping


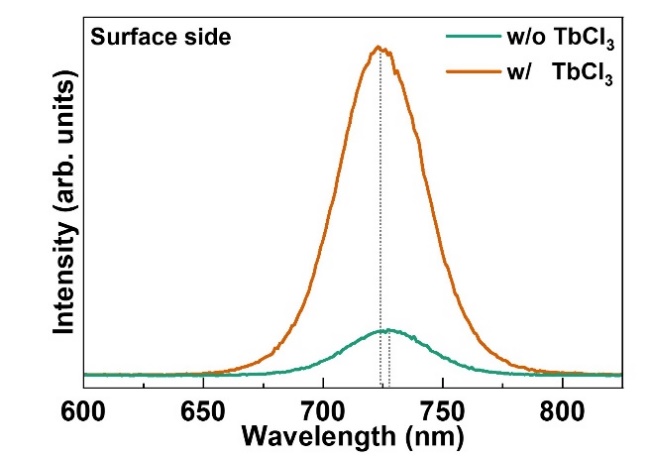


**Fig. S5** PL spectra from the surface side of CsPbI_3_ films prepared on Me-4PACz with and without TbCl_3_ doping


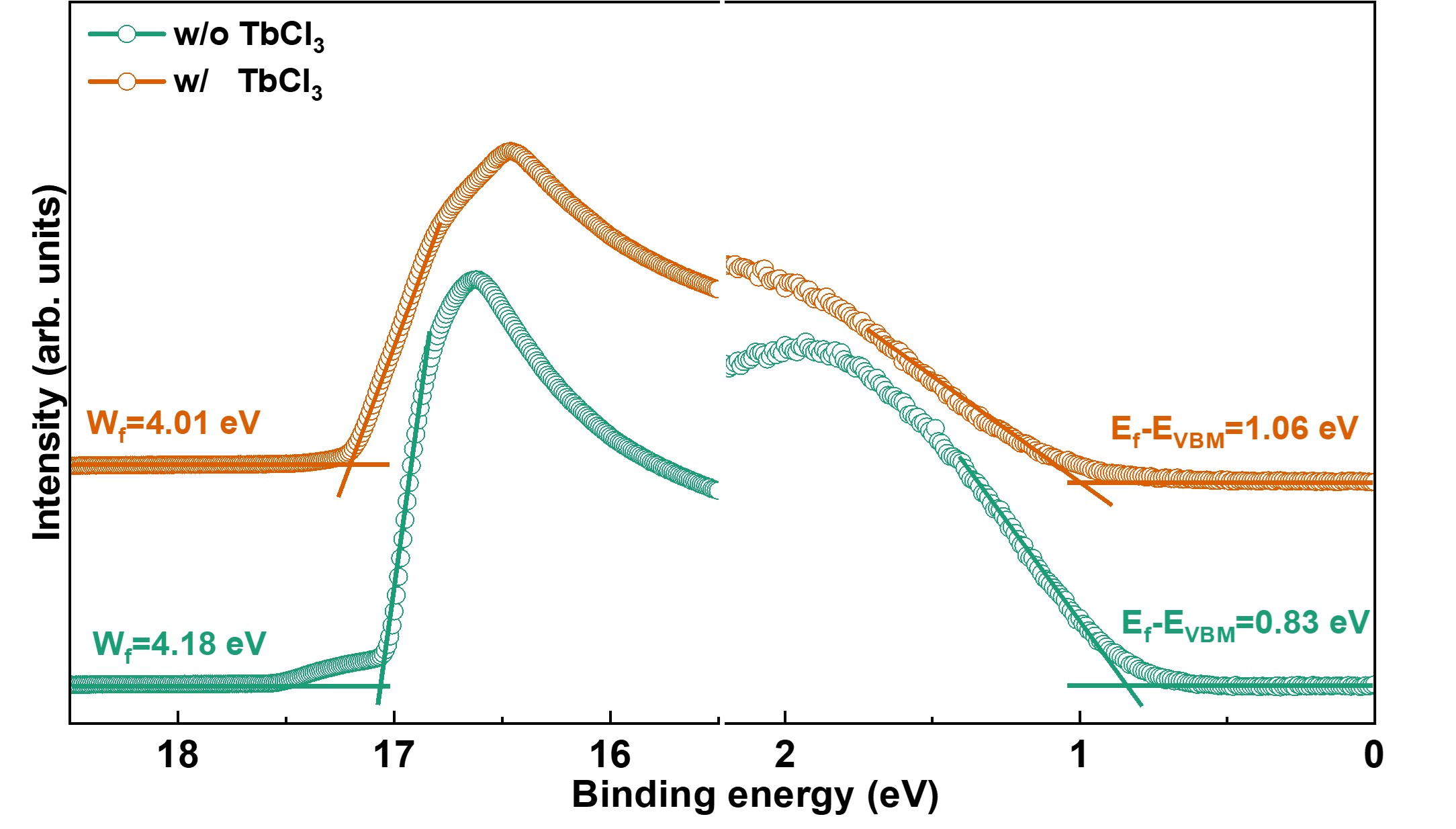


**Fig. S6** UPS spectra of Me-4PACz with and without TbCl_3_ doping


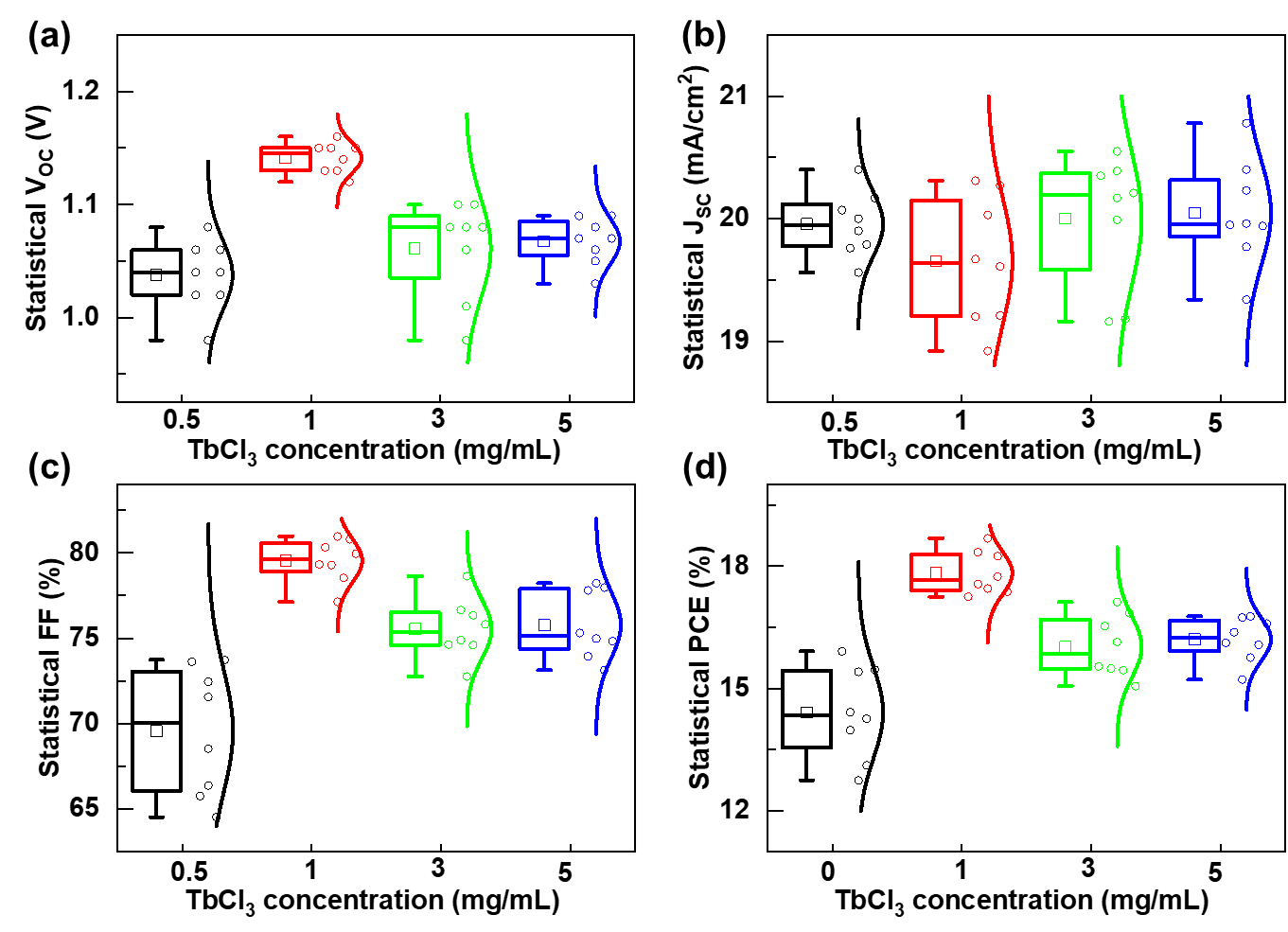


**Fig. S7** Statistical **a** V_OC_, **b** J_SC_, **c** FF and **d** PCE for CsPbI_3_ PSCs prepared on Me-4PACz with 0.5, 1, 3, and 5 mg/mL TbCl_3_ doping


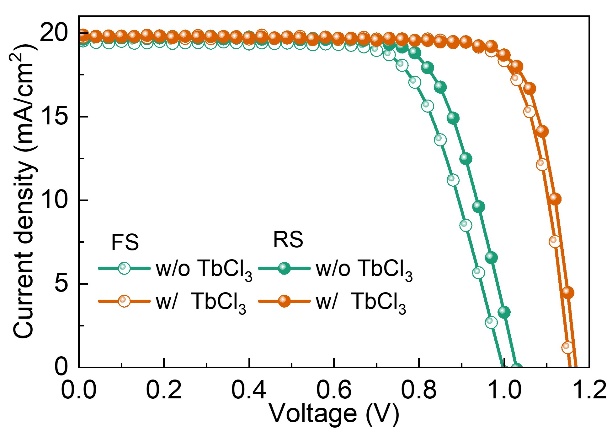


**Fig. S8** Light J-V curves recorded under forward voltage scan from -0.1 to 1.3 V and reverse voltage scan from 1.3 to -0.1 V for the champion CsPbI3 PSC prepared without and with TbCl3-doped Me-4PACz


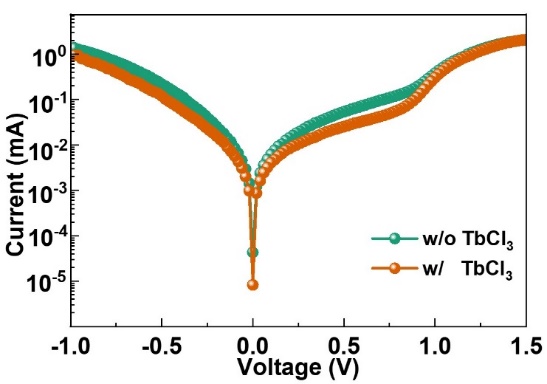


**Fig. S9** Dark J-V curves of CsPbI_3_ PSCs based on Me-4PACz with and without TbCl_3_ doping


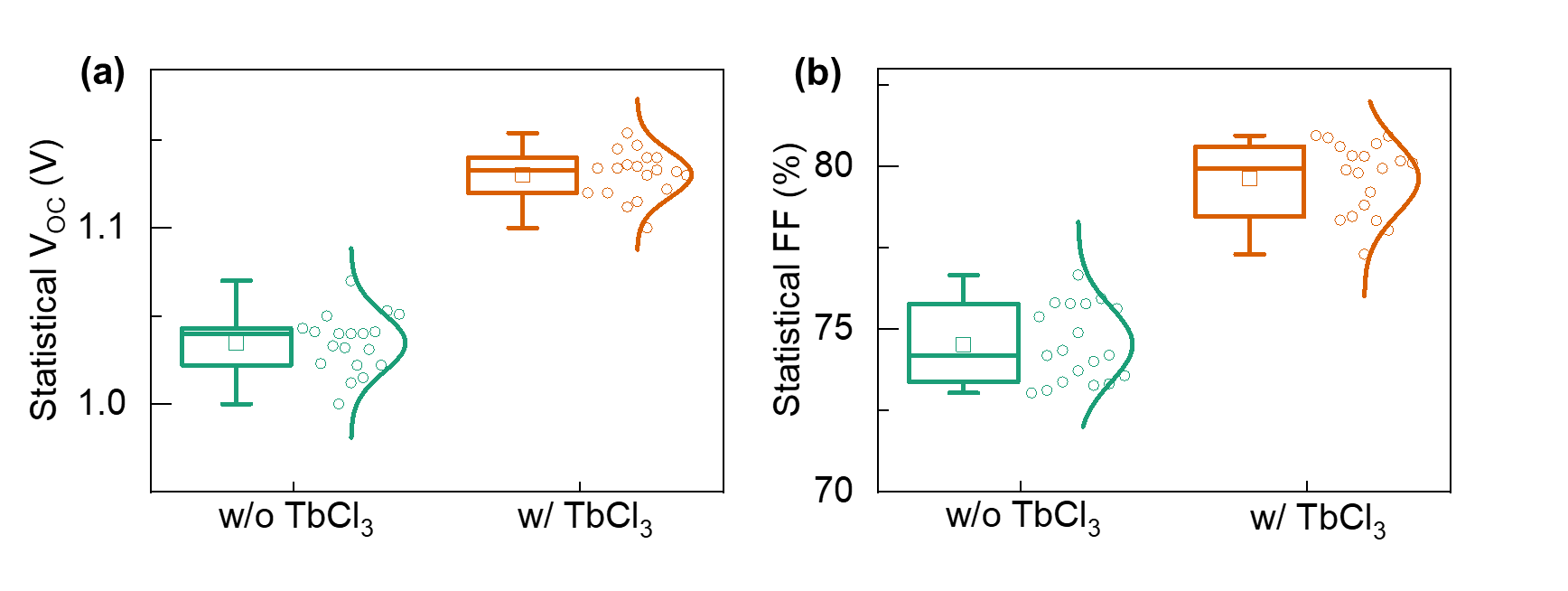


**Fig. S10** Statistical **a** V_OC_ and **b** FF for CsPbI_3_ PSCs prepared on Me-4PACz without and with TbCl_3_ doping


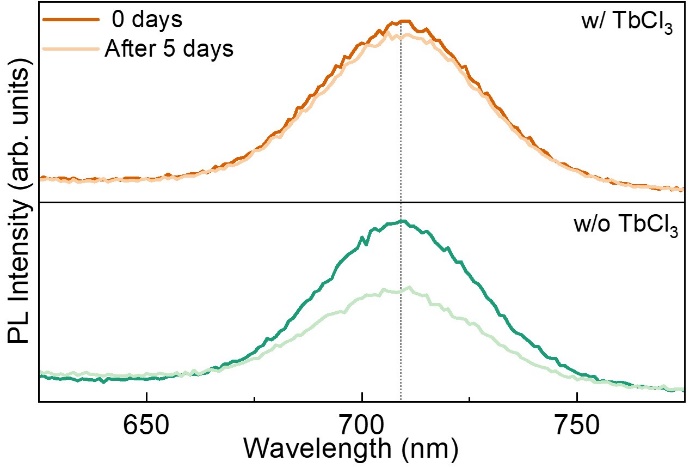


**Fig. S11** PL spectral of CsPbI_3_ films prepared without and with TbCl_3_-doped Me-4PACz after storing for 5 days in ambient air


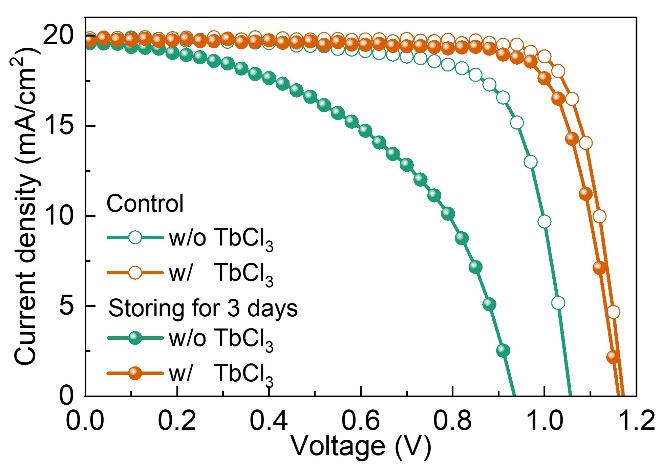


**Fig. S12** J-V curves of CsPbI_3_ PSCs fabricated without and with TbCl_3_-doped Me-4PACz after storing for 5 days in ambient air


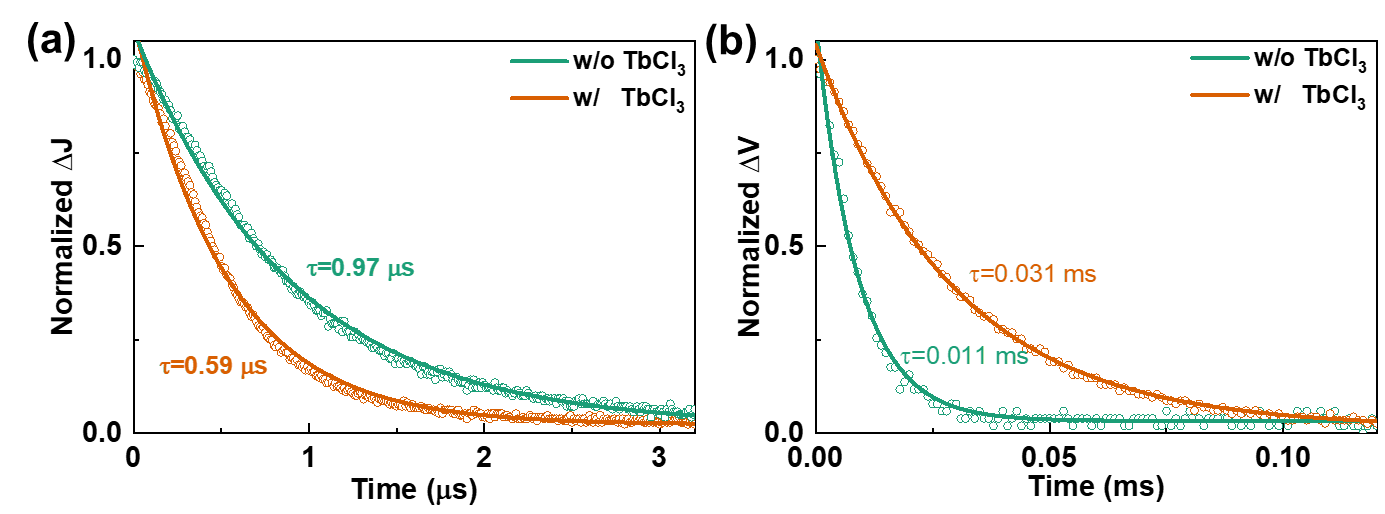


**Fig. S13** **a** TPC and **b** TPV curves of CsPbI_3_ PSCs based on Me-4PACz with and without TbCl_3_ doping


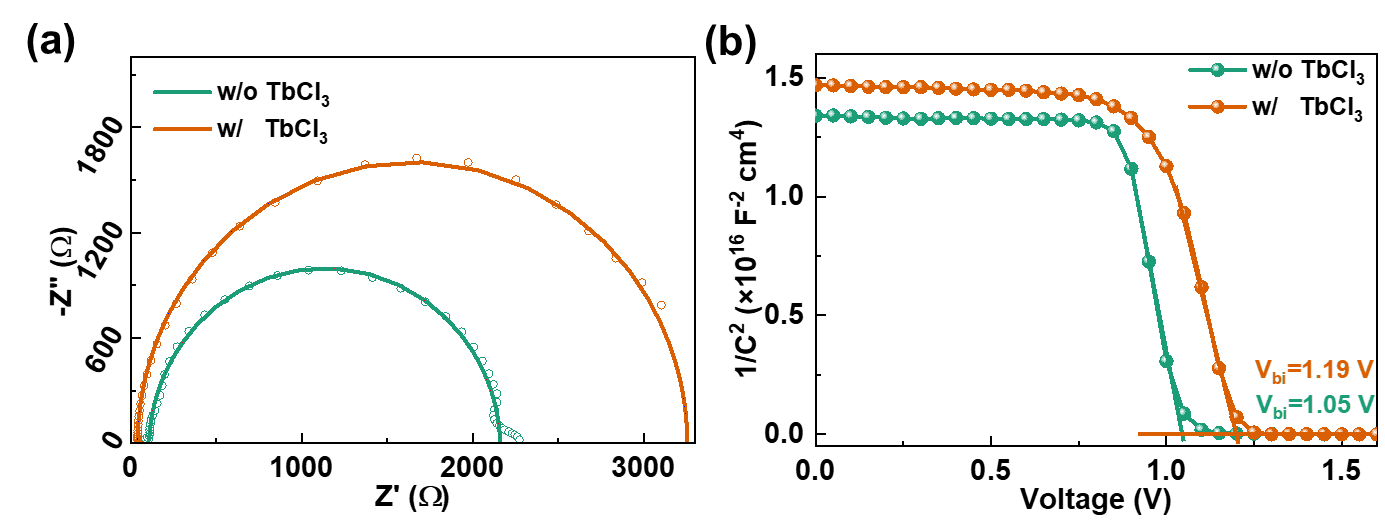


**Fig. S14** **a** Nyquist and **b** M-S plots of CsPbI_3_ PSCs based on Me-4PACz with and without TbCl_3_ doping

**Table S1** The fitting parameters of TRPL spectra for CsPbI_3_ films on Me-4PACz with and without TbCl_3_ doping

|  | **A_1_** | **τ_1_ (ns)** | **A_1_** | **τ_2_ (ns)** | **τ_ave_ (ns)** |
| --- | --- | --- | --- | --- | --- |
| w/o TbCl_3_ | 21.93 | 0.46 | 0.37 | 1.66 | 2.67 |
| w/ TbCl_3_ | 2.02 | 0.28 | 0.59 | 2.79 | 1.55 |

**Table S2** Photovoltaic parameters of the optimized CsPbI_3_ PSCs based on Me-4PACz with and without TbCl_3_ doping

| **Samples** | **V_OC_ (V)** | **J_SC_ (mA/cm^2^)** | **FF** | **PCE (%)** |
| --- | --- | --- | --- | --- |
| w/o TbCl_3_ | 1.017 | 19.9 | 0.758 | 15.34 |
| w/ TbCl_3_ | 1.162 | 20.12 | 0.799 | 18.68 |

**Table S3** Photovoltaic parameters of the semitransparent CsPbI_3_ PSC, the silicon solar cell before and after filtering, and the perovskite/silicon tandem device

| **Samples** | **J_SC_ (mA/cm^2^)** | **V_OC_ (V)** | **FF** | **PCE (%)** |
| --- | --- | --- | --- | --- |
| Si-Cell | 41.81 | 0.72 | 0.761 | 22.91 |
| Si-Filtered | 21.93 | 0.70 | 0.770 | 11.83 |
| Semitransparent PSCs | 19.75 | 1.11 | 0.802 | 17.57 |
| 4T TSCs |  |  |  | **29.40** |
| 2T TSCs | 18.56 | 1.75 | 0.783 | **25.44** |

**Table S4** Performance comparisons of the tandem solar cells based on all-inorganic perovskite materials

| **Device structure** | **All-inorganic materials** | **PCE (%)** | **Year** | **References** |
| --- | --- | --- | --- | --- |
| all-perovskite TSC | CsPbI_1.5_Br_1.5_ | 18.07 | 2022 | [S1] |
| all-perovskite TSC | CsPbI_2_Br | 19.61 | 2023 | [S2] |
| Perovskite/organic TSC | CsPbI_2_Br | 17.24 | 2020 | [S3] |
| Perovskite/organic TSC | CsPbI_2.1_Br_0.9_ | 18.06 | 2021 | [S4] |
| Perovskite/organic TSC | CsPbI_1.8_Br_1.2_ | 21.04 | 2022 | [S5] |
| Perovskite/organic TSC | CsPbI_2_Br | 20.2 | 2022 | [S6] |
| Perovskite/organic TSC | CsPbI_2_Br | 21.4 | 2022 | [S7] |
| Perovskite/organic TSC | CsPbI_2.2_Br_0.8_ | 22.43 | 2022 | [S8] |
| Perovskite/organic TSC | CsPbI_2_Br | 23.21 | 2023 | [S9] |
| Perovskite/organic TSC | Cs_0.99_Rb_0.01_PbI_2.2_Br_0.8_ | 23.07 | 2024 | [S10] |
| Perovskite/organic TSC | CsPbI_2.5_Br_0.5_ | 23.24 | 2024 | [S11] |
| Perovskite/organic TSC | CsPbI_2_Br | 22.34 | 2023 | [S12] |
| Perovskite/silicon TSC | CsPbI_x_Br_3-x_ | 22.95 | 2022 | [S13] |
| Perovskite/silicon TSC | CsPbI_2.85_Br_0.15_ | 25.31 | 2023 | [S14] |
| Perovskite/silicon TSC | CsPbI_2.85_Br_0.15_ | 27.27 | 2024 | [S15] |
| 2T Perovskite/silicon TSC | CsPbI_3_ | **25.44** | 2024 | **This work** |
| 4T Perovskite/silicon TSC | CsPbI_3_ | **29.40** | 2024 | **This work** |

**Supplementary References**

1. Q. Sun, Z. Zhang, T. Zhang, Y. Feng, A. Gu et al. Integrated 4-terminal all-inorganic perovskite tandem solar cell with open-circuit voltage exceeding 2.1 V for water splitting. ACS Energy Lett. **7**(12), 4215-4223 (2022). <https://doi.org/10.1021/acsenergylett.2c02262>
2. Q. Wen, C. Duan, F. Zou, D. Luo, J. Li et al. All-inorganic CsPb_1-x_Sn_x_I_2_Br perovskites mediated by dicyandiamide additive for efficient 4-terminal tandem solar cell. Chem. Engin. J. **452**, 139697 (2023). <https://doi.org/10.1016/j.cej.2022.139697>
3. K. Lang, Q. Guo, Z. He, Y. Bai, J. Yao et al. High performance tandem solar cells with inorganic perovskite and organic conjugated molecules to realize complementary absorption. J. Phys. Chem. Lett. **11**(22), 9596-9604 (2020). <https://doi.org/10.1021/acs.jpclett.0c02794>
4. X. Wu, Y. Liu, F. Qi, F. Lin, H. Fu et al. Improved stability and efficiency of perovskite/organic tandem solar cells with an all-inorganic perovskite layer. J. Mater. Chem. A **9**(35), 19778-19787 (2021). <https://doi.org/10.1039/D0TA12286F>
5. W. Chen, D. Li, X. Chen, H. Chen, S. Liu et al. Surface reconstruction for stable monolithic all‐inorganic perovskite/organic tandem solar cells with over 21% efficiency. Adv. Funct. Mater. **32**(5), 2109321 (2022). <https://doi.org/10.1002/adfm.202109321>
6. X. Gu, X. Lai, Y. Zhang, T. Wang, W. L. Tan et al. Organic solar cell with efficiency over 20% and V_OC_ exceeding 2.1 V enabled by tandem with all‐inorganic perovskite and thermal annealing‐free process. Adv. Sci. **9**(28), 2200445 (2022). <https://doi.org/10.1002/advs.202200445>
7. Y. Ding, Q. Guo, Y. Geng, Z. Dai, Z. Wang et al. A low-cost hole transport layer enables CsPbI_2_Br single-junction and tandem perovskite solar cells with record efficiencies of 17.8% and 21.4%. Nano Today **46**, 101586 (2022). <https://doi.org/10.1016/j.nantod.2022.101586>
8. Q. Yao, Y. M. Xie, Y. Zhou, Q. Xue, X. Xu et al. Dual sub‐cells modification enables high‐efficiency n–i–p type monolithic perovskite/organic tandem solar cells. Adv. Funct. Mater. **33**(8), 2212599 (2023). <https://doi.org/10.1002/adfm.202212599>
9. S. Q. Sun, X. Xu, Q. Sun, Q. Yao, Y. Cai et al. All‐inorganic perovskite‐based monolithic perovskite/organic tandem solar cells with 23.21% efficiency by dual‐interface engineering. Adv. Energy Mater. **13**(16), 2204347 (2023). <https://doi.org/10.1002/aenm.202204347>
10. S. S. Mali, J. V. Patil, J. A. Steele, M. K. Nazeeruddin, J. H. Kim et al. All-inorganic halide perovskites for air-processed “n–i–p” monolithic perovskite/organic hybrid tandem solar cells exceeding 23% efficiency. Energy Environ. Sci. **17**(3), 1046-1060 (2024). <https://doi.org/10.1039/D3EE02763E>
11. Y. Li, Y. Yan, Y. Fu, W. Jiang, M. Liu et al. Highly durable inverted inorganic perovskite/organic tandem solar cells enabled by multifunctional additives. Angew. Chem. Int. Ed., e202412515 (2024). <https://doi.org/10.1002/anie.202412515>
12. L. Liu, H. Xiao, K. Jin, Z. Xiao, X. Du et al. 4-Terminal inorganic perovskite/organic tandem solar cells offer 22% efficiency. Nano-Micro Lett. **15**(1), 23 (2023). <https://doi.org/10.1007/s40820-022-00995-2>
13. 13. S. Wang, P. Wang, B. Chen, R. Li, N. Ren et al. Suppressed recombination for monolithic inorganic perovskite/silicon tandem solar cells with an approximate efficiency of 23%. EScience **2**(3), 339-346 (2022). <https://doi.org/10.1016/j.esci.2022.04.001>
14. S. Wang, P. Wang, B. Shi, C. Sun, H. Sun et al. Inorganic perovskite surface reconfiguration for stable inverted solar cells with 20.38% efficiency and its application in tandem devices. Adv. Mater. **35**(28), 2300581 (2023). <https://doi.org/10.1002/adma.202300581>
15. S. Wang, H. Sun, P. Wang, X. Ge, Y. Li et al. Small molecule regulatory strategy for inorganic perovskite solar cells with 368 mV of V_OC_ deficit and its application in tandem devices. Adv. Energy Mater., 2400151 (2024). <https://doi.org/10.1002/aenm.202400151>
